# Proper Management of Appendicitis

**Published:** 1897-02

**Authors:** 


### THE PROPER MANAGEMENT OF APPENDICITIS.

Dr. J. W. Hickman, in *Medical News*, Nov. 21, gives it as his opinion that appendicitis is a purely surgical disease, and that the only safe treatment is an operation. He writes:

"Other therapeutic measures will avail in perhaps a majority of cases, at least in the first attack, or posssibly in the first few attacks. I know, however, that any other means of treatment will be marked by a mortality rate, and this must be true until we are able to separate catarrhal from suppurative cases. This, I repeat, can not be done at present. Then, why not operate at once in all cases? Such a course, in properly qualified hands, is as nearly absolutely safe as any operation can be. Suppose some cases are operated on that would have gotten over the attack without an operation? Better this than to run so grave a risk as we must encounter when we wait."—*American Med Review.*

### MEXICAN OBSTETRICS.

At the late Pan-American Congress, Dr. Capetillo, of Mexico City, read a paper saying dystocia from maternal causes was comparatively rare in Mexico, and especially so was that from narrowed pelvis in consequence of rickets or osteomalacia. Dystocia was sometimes caused in the native (Indian) women by reason of the great narrowness of the vulva and rigidity of the perineum in them as compared with women of Spanish blood. Dystocia sometimes occurs in consequence of the premature rupture of the bag of waters, resulting from the common use of the Montana fomentosa, or "Zoapatl." Placenta previa and procidentia are rather common causes of dystocia. —*Medical Record.*

